# Strategies for using mathematical modeling approaches to design and interpret multi-organ microphysiological systems (MPS)

**DOI:** 10.1063/1.5097675

**Published:** 2019-06-20

**Authors:** Jong Hwan Sung, Ying Wang, Michael L. Shuler

**Affiliations:** 1 Department of Chemical Engineering, Hongik University, Seoul 04066, South Korea; 2 Nancy E. and Peter C. Meinig School of Biomedical Engineering, Cornell University, Ithaca, New York 14853, USA; 3 Hesperos, Inc. Orlando, Florida 32836, USA; 4 Robert Frederick Smith School of Chemical and Biomolecular Engineering, Cornell University, Ithaca, New York 14853, USA

## Abstract

Recent advances in organ-on-a-chip technology have resulted in numerous examples of microscale systems that faithfully mimic the physiology and pathology of human organs and diseases. The next step in this field, which has already been partially demonstrated at a proof-of-concept level, would be integration of organ modules to construct multiorgan microphysiological systems (MPSs). In particular, there is interest in “body-on-a-chip” models, which recapitulate complex and dynamic interactions between different organs. Integration of multiple organ modules, while faithfully reflecting human physiology in a quantitative sense, will require careful consideration of factors such as relative organ sizes, blood flow rates, cell numbers, and ratios of cell types. The use of a mathematical modeling platform will be an essential element in designing multiorgan MPSs and interpretation of experimental results. Also, extrapolation to *in vivo* will require robust mathematical modeling techniques. So far, several scaling methods and pharmacokinetic and physiologically based pharmacokinetic models have been applied to multiorgan MPSs, with each method being suitable to a subset of different objectives. Here, we summarize current mathematical methodologies used for the design and interpretation of multiorgan MPSs and suggest important considerations and approaches to allow multiorgan MPSs to recapitulate human physiology and disease progression better, as well as help *in vitro* to *in vivo* translation of studies on response to drugs or chemicals.

## I. INTRODUCTION

Microphysiological systems (MPSs), also known as organ-on-a-chip, are engineered, microscale *in vitro* tissues that mimic aspects of human physiology. The advantages of MPSs come from the fact that they can provide a physiologically relevant tissue microenvironment, such as blood flow, 3D tissue matrix, cell-cell interactions, and chemical gradient.^1^ It is expected that development of more physiologically realistic *in vitro* platforms will help overcome some of the current challenges that the pharmaceutical industry is facing in drug development.^2^ Recent advances during the last decade in developing single microphysiological systems (MPSs) have led to interest in interconnecting single MPSs to realize multiorgan interactions.^3–5^ Although proof-of-concept studies on a multiorgan MPS, also often termed body-on-a-chip, have been published as early as in 2004,^6,7^ several challenges remain to create more advanced multiorgan MPSs that reproduce complex human physiology involving interactions between multiple organs.

**FIG. 1.**
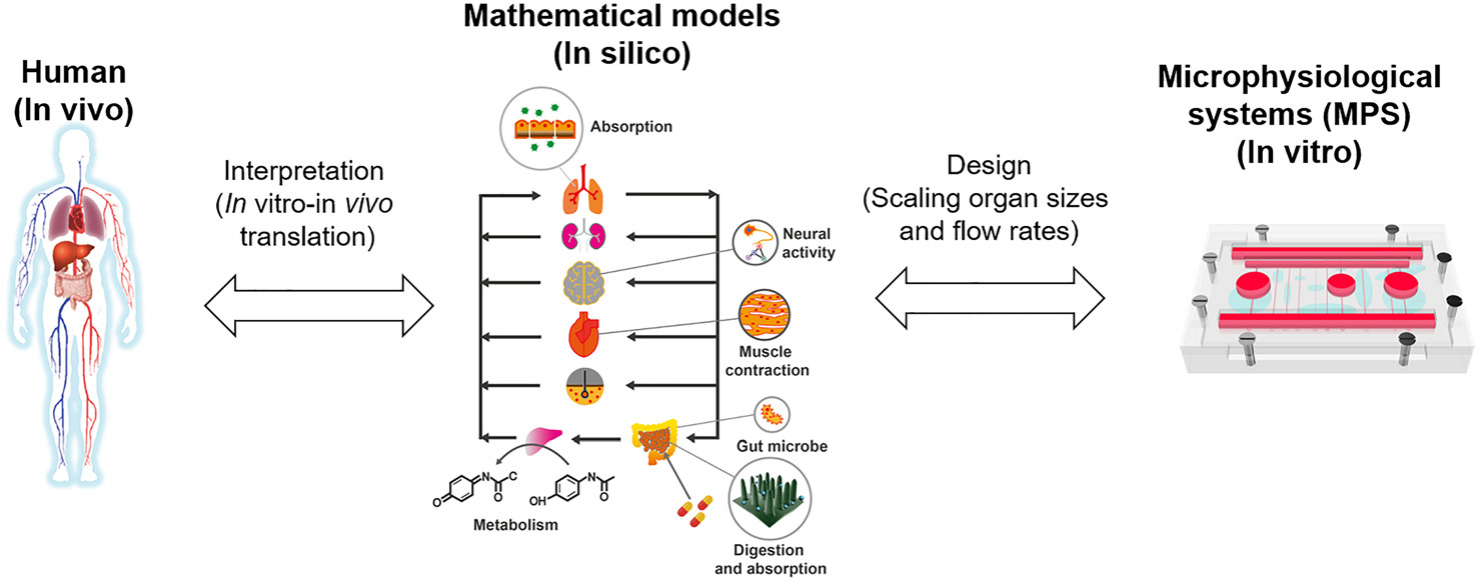
Relationship between *in vivo* (human or animals), *in silico* (mathematical models), and *in vitro* (MPS) platforms.

One of the challenges researchers are facing today is that as the number of organs in the system increases, the complexity of the system increases dramatically. The increased complexity of the multiorgan MPS gives rise to several issues: (1) how to determine appropriate ratios of sizes of different organs, as well as total medium (blood) volume, given that a “physiologically unrealistic” ratio between organs will often distort the nature of their interactions and make it difficult to determine appropriate flow rates between different organs, as well as the recirculating flow within the device and (2) how to translate the experimental results from multiorgan MPSs to prediction of *in vivo* responses in humans; for example, can we directly compare drug concentrations measured from a MPS with drug concentrations measured from human plasma samples, or would we need some kind of scaling factors to make a comparison? The first question is related to the issue of designing multiorgan MPSs, and the second question is related to the interpretation of MPSs. In addition, they are problems related to each other, because how the MPS is designed will affect how to interpret experimental data obtained from the system (Fig. 1).

The complexity of these problems—how to design a multiorgan MPS, so that it correctly reproduces human physiology, and how to interpret the MPS and translate experimental data to the *in vivo* case require a robust mathematical approach. Although the concept of MPSs is relatively new, mathematical approaches for designing and interpreting the MPS can be adapted from mathematical techniques that have been used in biological and pharmacological sciences, which have been developed and validated for several decades. Here, we review the current progress in application of mathematical techniques to multiorgan MPSs. We will summarize how traditional mathematical models such as allometric scaling and pharmacokinetic (PK) models are adapted and applied to MPSs. Although we are only at the beginning stage in this area, we are beginning to see more refined and robust approaches, often accompanied by validation with experimental data. We intend this review paper to be a summary of recently reported methodologies and evaluation of strengths and weaknesses, as well as their perspectives on future directions.

## II. DESIGNING MPSs

### A. Considerations for designing MPSs

Multiorgan MPSs aim to recapitulate the complex and dynamic interactions among tissues and organs in the human body. In addition to strategies for re-creating tissue-like structures and functions as in single-organ MPSs, special design considerations are needed for multiorgan MPSs to appropriately reflect the *in vivo* organ-organ relationships and to reproduce physiologically relevant multiorgan interactions.

#### 1. Common media and multiorgan interconnections

Fluidic connections among organ modules are often required to recreate cross-organ interactions. In currently developed models, interorgan communications are mainly achieved through a systemic fluid pool (common medium) that interconnects different organ modules, mimicking the circulating blood. Such a systemic fluid pool can potentially transport nutrients, soluble ligands (growth factors, cytokines, hormones, etc.), cell metabolites, pharmaceutical drugs, and cellular components (exosomes, miRNA, mRNA, tumor DNA, and even circulating cells). It constantly interacts with the local microenvironment of different tissues and mediates organ crosstalk.

Developing a common medium to support the maintenance of phenotypes and functions of all organs in the MPS is critical and not trivial. Complementary strategies have been demonstrated and often combined to achieve an optimal multiorgan performance. Several multiorgan MPSs with a liver module chose liver culture media as the common medium to ensure hepatic functions in the coculture system, due to the liver's high metabolic demand and its central role in drug metabolism.^8–13^ Some have mixed organ-specific media for all organs in an equal ratio as the common medium to meet the needs (such as essential growth factors) of all organ modules to a certain degree.^14,15^ Growth factors with contradictory effects on different organs which are not suitable for global administration have been supplied locally to satisfy the special needs of specific organs.^14^

In addition to the formulation of a common medium, the way it interconnects different organ modules can greatly affect the effectiveness of a common medium in supporting multiorgan functions and mediating interorgan communications. Different fluidic interconnection platforms have been thoroughly reviewed previously,^5^ which mainly include (A) static microscale platforms; (B) single-pass microfluidic platforms; (C) pump driven, and (D) pumpless recirculating microfluidic platforms. Compared to static fluidic integration relying on passive diffusion, dynamic microfluidic interconnections enable the establishment of controllable and reliable biochemical gradients that drive mass exchange between the systemic fluid perfusion and local tissue microenvironments. The architecture of the interconnecting fluid networks can have a large impact on organ crosstalk in multiorgan systems. Single-pass microfluidic systems, where the common medium is perfused through all organ modules in an open-loop, sequential manner, are usually easy to set up, yet the interorgan communications are mostly unidirectional lacking feedback loops from downstream organs to the upstream ones. The recirculating microfluidic systems provide closed loop perfusion that better mimics the blood circulation and facilitates reciprocal communications among organs. Pumpless recirculating microfluidic platforms using reciprocating gravity-induced flows for recirculation have demonstrated low-cost, hassle-free, and long-term (up to 4 weeks) maintenance of several multiorgan MPSs,^13,16–18^ while providing similar pharmacokinetic (PK) profiles as the pump-driven continuous unidirectional perfusion.^19^ “UniChip,” a recent breakthrough in pumpless platforms, has achieved continuous unidirectional perfusion that enables incorporation of shear stress sensitive tissues (vasculature, lungs, kidneys, etc.) into a pumpless system.^20^ The UniChip design also promises incorporation of circulating cells (circulating tumor cells, immune cells, etc.) into recirculating MPSs, which has been challenging using pump-driven platforms and would allow one to model live cell-mediated interorgan communications, such as distant cancer metastasis and immune responses. Moreover, interconnecting organs using serial, parallel, or combined network architecture, as well as the relative location of different organs in the network are also important design considerations. A single-loop, serial systemic interconnecting network has been used in a majority of current multiorgan models due to its simplicity. While allowing for organ crosstalk, such a fluid network has a built-in limitation of applying the same flow rate for all organs. It is possible to arrange the location of organ modules to mimic the physiological process better. For example, a gastrointestinal (GI) tract module in close vicinity to a liver module allows simulation of the first-pass effect in which pharmaceutical drugs are metabolized by the liver model immediately after GI tract absorption before reaching the systemic circulation.

#### 2. Physiological relevance of multiorgan interactions

While fluidic interconnections among organ models allow for organ-organ interactions *in vitro*, whether those interactions represent human responses depends not only on single-organ functions reproduced *in vitro* but also on the physiological relevance of organ-organ relationships imbedded in the system design. It is not realistic to build a perfect “mini human” that represents every aspect of the human body, neither it is the goal. It is therefore critical to identify most of the essential parameters affecting the on-chip simulation of a multiorgan MPS based on its target applications. Most biochemical reactions and biological responses are concentration dependent. Design parameters, such as relative organ sizes, flow distribution among organs, and liquid-to-cell (LoC) ratios, can significantly affect the global (systemic) and local concentrations of key molecules and cellular components that mediate organ crosstalk. They are thus important design considerations for multiorgan MPSs.

Derivation of on-chip organ sizes from physiological values requires proper scaling, which will be discussed in detail in Sec. II B. The organ sizes of different organs in a multiorgan MPS may also carry different weights in design for recapitulating the dynamic organ interactions *in vitro*. Organ models, based on their major roles in the organ-organ interactions, can be viewed as either “source organs” that generate or significantly affect the bioavailability of molecules and cellular components mediating the organ crosstalk, or “target organs” that can have significant responses to these mediators, or both. The size of a source organ (i.e., the surface area of a barrier tissue or the volume of a parenchymal tissue) can have a big impact on the extent to which the organ model affects the composition of the systemic pool. For example, the relative sizes of the major organs involved in ADME—drug absorption (e.g., GI tract), distribution (e.g., adipose tissues, the blood-brain barrier), metabolism (e.g., liver), and excretion (e.g., kidneys)—would have much greater impact on the on-chip pharmacokinetics than target organs (e.g., cardiac tissues for cardiotoxicity). In particular, the liver tissue volume or hepatocyte number is a key design consideration due to the critical role of the liver in metabolism. Liquid per hepatocyte is often used as a measure for the metabolic burden in the system. Such practical considerations combined with scaling rules would help create physiologically relevant organ-organ relationships *in vitro*.

The flow rate and flow distribution among organs affect organ perfusion rates and molecules' resident time in organ models, which could alter the kinetics of biochemical reactions. For example, the perfusion rate of the liver model affects not only the nutrient supply for maintaining hepatic functions but also the first-pass metabolic rate, and thus the bioavailability of drugs and their metabolites in the systemic circulation. The LoC ratios in the MPS directly affect the concentrations of many crosstalk-mediating molecules and cellular components. A large LoC ratio will lead to overdilution of these mediators including drug metabolites and consequently incorrect PK data, mechanistically different responses, or failure to detect certain drug effects through organ-organ interactions. Therefore, design parameters affecting LoC ratios should be taken into particular consideration. By far, it has been very challenging to establish physiologically realistic ratios of LoC volumes at both local and systemic levels.^5^ Strategies to lower LoC ratios include combining 3D tissue culture and microfabrication to lower local LoC ratios as well as minimizing dead volume in the circuits (e.g., debubbler, interconnecting tubing, reservoirs) to decrease LoC ratios at systemic levels.^5^

Additional practical factors may also affect the dynamic profiles of key molecules and cellular components in multiorgan interactions. Different platform materials can vary significantly in their absorption and adsorption rates for different drugs and cell metabolites. It is thus important to minimize or at least thoroughly characterize such sorption for the drugs and metabolites of interest. Incorporation of on-chip biosensors, such as microelectrodes, microcantilevers, or biochemical sensors, is also likely to increase the overall fluid in the system, and thus LoC ratios. Balancing the need for on-chip functional analysis and that for maintaining close to physiological LoC ratios should be factored into design considerations.

### B. Scaling methods

A multiorgan MPS can be thought of as essentially a simplified and miniaturized version of the human body. This requires a well-thought strategy for “scaling,” specification of organ sizes, and operating conditions (flow rates in each organ modules, the total volume of media in the system, etc.) for the MPS, to successfully reproduce essential physiological functions of interest, as well as the response to drugs. There is still no widely accepted consensus on the optimal method of scaling an MPS, and it is likely that the choice depends on the objective of a study using the MPS. Several scaling methodologies have been proposed, often accompanied by proof-of-concept experimental studies, to validate the proposed scaling methods. Here, we summarize recently reported approaches for scaling multiorgan MPSs and how different scaling methods “perform” in terms of physiological relevance.

#### 1. Direct scaling

**FIG. 2.**
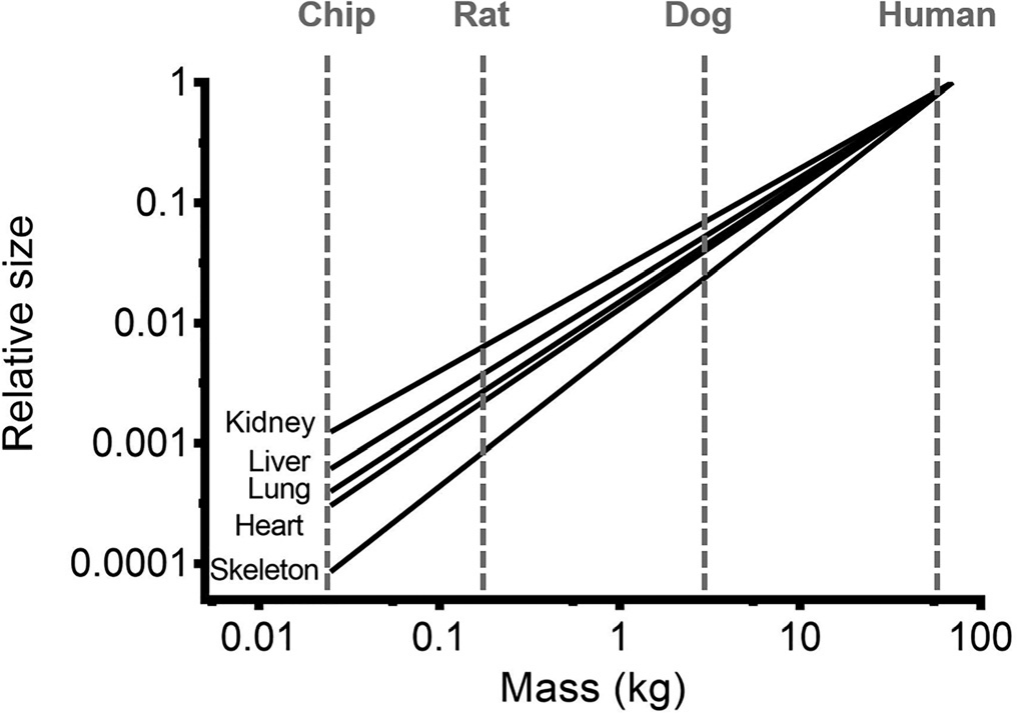
Relative sizes of various organs in species of different sizes. For each organ, its size was set to 1 for human. The microscale chip (MPS) scales down even further than most animals.

The most obvious method for scaling down various organs comprising the human body is to directly scale down each organ proportionally. Anatomical data for organs in the human and animals are easily accessible.^21^ To directly scale the human body to microscale size, one can simply divide the size of each target organ by the miniaturization factor. This method is straightforward and simple to apply. However, directly scaling different organs with same factors is likely to result in distortion of appropriate relationships between the organs, as it is known that different organs scale differently, as can be seen in Fig. 2. Wagner *et al.* developed a two-organ MPS (liver and skin), by combining microtissues and skin biopsies with the size of 1/100 000 of the biomass of their original human organ counterparts.^11^ The two tissues were maintained for 14 days while being exposed to fluid flow. The exact dimensions of the culture compartments for the liver and the skin were not specified in the paper, but it could be deduced from the description in the paper that they were of sizes of several millimeters. The flow rate of the recirculated media was 40 *μ*l/min with 600 *μ*l of total media volume. This implies significantly slower recirculation time compared to the blood recirculation in the human body (15 min in the chip vs approximately 5 min in the human body, calculated by dividing the total blood volume by the cardiac output, which are 5.2 l and 5.6 l/min for a 70 kg human, respectively^21^). Regarding the fluid-to-tissue ratio, which is another important scaling factor to be considered, the authors stated that one of their design principles was to minimize the fluid-to-tissue ratio, to prevent the dilution of signaling molecules due to undesirably large volume of fluid (media). The authors presented the evidence of crosstalk between the liver microtissue and skin biopsy by measuring the time-dependent concentration of albumin. The measurement results suggested that the albumin consumed by the skin was produced by the liver. This result implies that a crosstalk between the liver and the skin was established successfully, and a state similar to an equilibrium of albumin production in the liver and consumption in the skin was established between the two organs. However, the physiological relevance of the measured albumin concentration, or consumption/production rates to the corresponding values in the human body, was not examined.

Earlier work by the same authors describes assumptions when they were designing the multiorgan MPS, but the focus was on establishing sufficient nutrients and oxygen supply to the tissue culture compartments, rather than the kinetics of crosstalk between different compartments.^22^ In a more recent paper, the authors reported a four-organ MPS containing intestine, liver, skin, and kidney equivalents in the chip.^8^ The authors also mentioned in the paper that the chip was designed such that the fluid-to-tissue ratio was low, although the exact ratio was not specified. The tissue viability in the chip was verified by lactate dehydrogenase (LDH) activity, and the glucose balance in the chip was verified for 28 days. Expression of major genes for each organ was examined for 28 days. These results successfully demonstrated that the four tissues were viable and functional during the 28-day culture period. However, this paper did not provide any evidence of crosstalk between the organs, and it is difficult to judge if the design of the chip was mimicking physiologically relevant crosstalk between the four organs.

#### 2. Residence-time based scaling and derivation of parametric design criteria

Shuler *et al.* have published a series of pioneering papers on multiorgan MPSs aimed at reproducing the interaction between organs.^3–5^ In one of the earlier papers, the working principles for designing a multiorgan MPS were described.^6^ The following four constraints were used when designing a three-chamber MPS (termed microscale cell culture analog in the paper).

(1) The ratio of the chamber sizes and the liquid residence times in each compartment reflects the physiological values in the human body.
(2) Each chamber should have a minimum of 104 cells.
(3) The hydrodynamic shear stress should be within physiological values.
(4) The liquid-to-cell ratio should be close to the physiological value of 1:2.

Among the mentioned four constraints, the first and the last constraints are directly related to the issue of scaling. The first constraint ensures that each organ is exposed to chemical cues for the same amount of time that the same organ would be exposed in the human body. The last constraint ensures that the chemical cues generated from the cells in the MPS are not diluted to an extent where they would not exert any observable effect.

This method, which we will term residence-time based scaling in this paper, is simple, but captures the essence of reaction kinetics within the organ tissues and organ compartments in a multiorgan MPS. Theoretically, abiding by these two constraints will allow recapitulation of the kinetics of reactions, that is, generation and consumption of molecules, by the cells in a quantitative sense. Based on this approach, several multiorgan MPSs have been designed and tested. For example, drug mixtures for treating multidrug resistant (MDR) cancers were tested in a four-organ MPS (liver, bone marrow, uterine cancer, and MDR variant of uterine cancer).^23^ A physiologically based pharmacokinetic (PBPK) simulation was used to compare the concentration profiles of a drug [doxorubicin (DOX)] and its metabolite [doxorubicinol (DOXOL)], which provide an important insight into whether the MPS is physiologically realistic. A similar approach was used to design a three-chamber MPS (liver, tumor, and bone marrow) for testing the pharmacokinetics (PK) and efficacy of anticancer drugs.^19^ In this paper, a pharmacodynamic (PD) model was added to a PK model to account for the cell-killing effect of an anticancer drug, which allowed the authors to simulate the time-dependent changes of cell viability in response to drugs. As illustrated in these examples, the use of PBPK modeling techniques is important in the design and interpretation of multiorgan MPSs, which will be discussed in more detail in Sec. III.

For the residence-time based scaling approach to be successful, each organ module should have activity or capacity comparable to that in the human body. Taking the liver as an example, if one can presume that the liver cells cultured within a MPS have intrinsic metabolic activity comparable to the hepatocytes in the human liver, and the liquid-to-cell ratio is sufficiently low so that it will not cause unnatural dilution of the generated molecules, the concentration of the metabolite produced by the liver cells in a MPS will be similar to that in the human body. A potential limitation of this approach is that it does not account for the mass transfer limitation within the tissues, which can occur frequently in a real situation but was not considered by implicitly taking a well-mixed assumption for each organ.

In a recent paper, Shuler and Abaci proposed a more refined approach based on the principles of PBPK modeling and derived parametric criteria for designing a multiorgan MPS.^24^ The authors defined two different platforms, *μ*Organs-on-a-chip (*μ*OOC) and *μ*Human-on-a-chip (*μ*HOC), which can be involved in different stages of drug development. The *μ*OOC is intended for early phases of drug development as a replacement to animal models. The primary goal of *μ*OOC is to estimate or validate appropriate parameters for a human PBPK model, and accurate reproduction of human PK is not required. The design criterion for *μ*OOC is that the steady-state concentration values of intrinsic metabolites, such as glucose, oxygen, and amino acids, in each organ should mimic the corresponding values in the human body. On the other hand, the *μ*HOC is intended for more advanced stages of drug development and should be able to directly mimic human PK. The design criterion for *μ*HOC is that the concentration profiles of a drug in the blood and tissues should mimic those in the human body. The main difference between *μ*OOC and *μ*HOC is that whereas *μ*OOC allows only the extraction of PBPK parameters, the *μ*HOC allows direct simulation of drug concentration profiles in the human body. Based on the macroscopic mass balance equations depicting these criteria and steady-state assumption, the authors were able to derive a general parametric equation for designing a *μ*OOC. Due to the large number of parameters, multiple solutions will exist for the derived equation, but a reasonable solution can be deduced based on human body parameters and operating considerations for the *μ*OOC. This work provides a quantitative and practical foundation for designing a multiorgan MPS, which can be potentially utilized in the drug development process. As is mentioned, this design equation can give multiple solutions, and it is probably necessary to refine the approach further to obtain more determinate solutions, by introducing additional constraints.

#### 3. Allometric scaling

Allometry is a study of relationship between the body size and various physiological parameters, and has a long history of more than a century since the first description.^25^ It is based on the idea that there is a governing law that dictates various physiological parameters depending on the size of organisms, which can be applied across species of a wide range of sizes. Allometric scaling laws can correlate the mass of organisms with physiological parameters, such as the metabolic rate,^26^ the heart rate, and the blood flow rate.^27^ The allometric scaling law is expressed by a power law equation with an exponent “b.” For example, the metabolic rates in many organisms follow an allometric scaling law with an exponent value of 3/4.

$$\text{Metabolic}\;\text{rate}={\;\text{aM}}^{3/4},$$ (1)

where a is a constant and M is the body mass. It has been proposed that the metabolic rate follows the allometric scaling law because nutrients are supplied to tissues through a fractal distribution network.^27^

Since a multiorgan MPS can be considered as a “miniaturized,” or “microscale” human body, application of allometric scaling laws to estimate the key physiological parameters of the MPS seems natural. Ahluwalia *et al.* published a series of papers on utilizing allometric scaling laws to design multiorgan MPSs of murine hepatocytes and human umbilical vein endothelial cells (HUVEC),^28^ primary rat hepatocytes and adipose tissues,^29^ adipose tissues, endothelial cells, and hepatocytes.^30^ The authors concluded that to elicit meaningful responses from a multiorgan MPS, the system requires (1) cell numbers and ratios, which enable appropriate physiological-like interactions and (2) flow rates that do not cause shear stress-related damage to cells and allow adequate residence times in each compartment to enable the compartment to process metabolic signals, while permitting an adequate oxygen supply through convection.^31,32^

In a more recent paper, Ahluwalia *et al.* proposed two different allometric scaling laws (the cell number scaling method and the metabolic and surface scaling method) and applied it to a two-organ model of hepatic-vascular crosstalk.^33^ In the metabolic and surface scaling method, organs are scaled differently depending on whether their main function is a volume-mediated process (such as metabolic conversion by hepatocytes) or surface-mediated process (such as distribution through endothelium). In the case of the liver, which is treated as volume-mediated, the basal metabolic rate (BMR) per cell hepatocyte is calculated from human parameters. Assuming that the same per cell BMR is maintained in the MPS and the fraction contribution of BMR by the liver (which is 27% in the human body) is also the same, the authors could calculate the total BMR of the MPS, if the number of hepatocytes in the system is fixed. Once the total BMR of the MPS is obtained, the authors used an allometric scaling law relating the body mass and the total BMR to determine the “body mass” of the MPS, which was calculated to be 1 mg. Having obtained the body mass, the allometric approach could be used again to calculate the vascular surface area. In the case of the second approach, the cell number scaling method, the fractions of total body weight by specific organs are considered (6.28% for vascular endothelial tissues and 2.6% for hepatic tissues in human), which can be used to calculate the ratio of the body's endothelial mass to the hepatic mass. Then, the hepatic-vascular MPS can be designed to maintain the ratio of endothelial to hepatic mass. Obviously, these two methods result in different MPS configurations, that is, cell numbers and chamber sizes. In some cases, unrealistic configurations may be deduced, wherein adjustment of parameters or additional assumptions are required for practicability.

The authors compared the two scaling methods by measuring the key markers related to carbohydrate, fat, and hepatic metabolism. Overall, the MPS designed with the cell number-based scaling method showed more physiologically realistic homeostasis values. It is unclear why one scaling method performed better than the other. The cell number-based scaling method considers the ratio of cell number between two organs and the metabolic and surface scaling method considers the ratio of the metabolic rate of two organs. It seems that at least for reproducing the homeostasis of glucose and fat metabolism, scaling based on the ratio of cell numbers is more appropriate. However, it should be noted that the optimal scaling method can be different for different purposes of study. For example, if one was to develop a multiorgan MPS for reproducing the PK of drugs, a different conclusion could have been drawn.

Moraes *et al.* proposed a “metabolically supported functional scaling” approach, which is based on the assumption that cells on the chip can be induced to maintain *in vivo* cellular basal metabolic rates by limiting the resources.^34^ The main principle of this approach is also based on allometric scaling of the metabolic rate, but the authors' approach complements the traditional allometric scaling by ensuring that the underlying prerequisite for allometric scaling is maintained in the MPS, that is, cells in the chip show per cell basal metabolic rates that would be observed *in vivo*. This prerequisite is achieved by limiting the nutrient supply to the cells, which causes cells *in vitro* to show basal per cell metabolic rates of cells *in vivo*. This is a notable observation that could complement the traditional allometric scaling approach, since cells cultured *in vitro* are generally exposed to excess nutrients and show different metabolic rates from their *in vivo* counterparts.^35^

An example of designing a two-organ MPS with adipose and vascular compartments was illustrated by the authors of Ref. 34. They ran a series of experiments using adipocytes in different configurations, that is, in dispersed spheroids and intact spheroids, which showed different mass transfer rates. One interesting observation made by the authors were that the structure of the microengineered tissue on the chip affects the scaling relationship. When the glucose uptake by adipose tissues in two different structures (dispersed vs intact spheroid) was compared, dispersed cells showed significantly higher glucose uptake, due to the difference in mass transport. This result clearly shows that when scaling multiorgan MPSs, the type of microtissue on the chip (for example, 2D monolayer, spheroids, cell suspension, 3D constructs, etc.) is important. In an approach to scale the two-compartment MPS, the authors first classify organs as being functionally 3D or functionally 2D. This is a similar concept to the one introduced by Ahluwalia, who classified the organ functions by the parameter whether they are volume-mediated or surface-mediated. Functionally 3D organs are scaled with their volume and functionally 2D organs are scaled with their surface area. The authors showed an example calculation of scaled mass and flow rate and the proposed chamber dimensions for 2D and 3D organs.

This approach makes good sense for designing a multiorgan MPS for studying glucose metabolism or metabolic diseases, such as glucose metabolism by adipocytes as illustrated in the paper. But similar to the traditional allometric scaling approach, it needs to be validated whether this approach can be useful for other purposes, for example, reproducing a PK of drugs. Also, in some cases, there can be some obscurities in determining whether a specific organ is functionally 2D or 3D. For example, the liver performs a metabolic function, which can be deemed 3D, but also performs secretory functions, which may be deemed 2D. Another notable challenge with this approach is that it can often result in physically impossible design dimensions, for example, unrealistically large organ volumes or flow rates. In this case, a further assumption or simplification may be required.

Wikswo *et al.* provided discussions on the pros and cons of different allometric scaling methods with very extensive and detailed example calculations of each method.^36^ The calculation of organ sizes by direct application of the allometric scaling law showed that allometric scaling down to microscale does not result in valid parameters for a multiorgan MPS. A second approach, termed by the authors as “interconnected histological sections” takes into account of the fact that cells in the chip may not exhibit the same physiological functions as *in vivo*, and the perfusion rate is simply determined by the number of cells in the system. As noted by the authors, this approach is likely to be valid only for recapitulating a subset of an organ's functions. Another approach proposed by the authors, termed “functional scaling,” first defines the major function of each organ. For example, heart: volume pumping; lungs: gas exchange; liver: metabolism; kidneys: molecular filtering and transport; and brain: blood-brain barrier function and synapse formation. After specifying the functional parameter for each organ, the multiorgan MPS can be scaled (iteratively), so that each organ meet the specific functional parameters, while keeping the constraints imposed by other factors, such as physical architecture of the device, materials, and available cells. The authors illustrate the example of functional scaling of individual organs, with extensive calculation examples and references, by defining about 250 anatomical and functional parameters.

This approach is more refined than the traditional allometric scaling method and mathematically more robust. However, similar to the case of metabolically supported functional scaling by Moraes *et al.*, there are some obscurities related to how to define the major function of an organ with more than one function. This can make it difficult to create an organ with more than one function. Along the same lines, an MPS designed for studying metabolic diseases may not work as an MPS for studying the PK of drugs. In some cases, it may be difficult to quantitatively define the functional parameters, as some of organ's physiological functions can only be defined qualitatively, rather than quantitatively. For example, it is difficult to define the neuronal activity in the brain or response of immune systems with a simple numerical parameter. In such cases, reduction of organ functions may be inevitable. In the example calculation illustrated by the authors, they limit the complex functions of the brain to the neurovascular unit (NVU) and the blood-brain-barrier (BBB), focusing more on the ADME studies. This approach can be justified by limiting the purpose of the MPS to a specific purpose only, rather than treating it as fully functional organs. In addition, the limited current knowledge about the physiology of organs often makes it difficult to define parameters.

#### 4. Multifunctional scaling

One of the important limitations of the allometric scaling method is that organs often possess multiple functions. In addition, there can be cases where realization of one organ function collides with the realization of different organ functions. For example, making the oxygen transfer in the lung compartment realistic might prevent recapitulating the hepatic conversion in the liver compartment. Another limitation is that often the parameters derived from the scaling method result in physically impossible configuration, as seen in previous examples. The facts that organs often carry out multiple functions and scaling need to be done for multiple organs implies that scaling a multiorgan MPS requires a systemic approach, or multivariate optimization.^37^

A mechanistic understanding of the processes that drugs go through inside a multiorgan MPS can help with the design of the system. Lee *et al.* reported a two-organ MPS (gut-liver) for reproducing the first-pass metabolism of orally administered drugs. Using paracetamol as a model drug, the authors compared the measured drug concentration profiles with the PK profile of the drug in humans from literature.^38^ Measured PK profiles in the MPS showed considerably slower clearance compared to the known PK in humans. A PK model of the two-organ MPS was constructed first with the original design parameters. Then, the design parameters were scaled based on the organ size (liver) and the absorptive surface area (gut), and the modified design parameters were tested by simulation of a PK model. The authors were able to conclude that a larger absorptive surface area and a higher hepatic conversion rate were necessary to achieve a more realistic PK profiles, and derive optimized the design parameters for the MPS.

In a recent paper, Maass *et al.* illustrated the use of an optimization approach, using a mechanistic model and by specifying multiple objective parameters.^39^ Two multiorgan MPSs were tested, gut-liver and gut-liver-kidney MPSs, for studying the PK of drugs after oral administration. First, the multifunctional scaling algorithm defines the objective function as a weighted squared difference between a model outcome and the corresponding measurements

$$\mathrm{Objective}\;\mathrm{function}=\text{min}{\left(\frac{\mathrm{prediction}-\mathrm{observation}}{\mathrm{prediction}}\right)}^{2}.$$ (2)

In the example in the paper, the objective function would use the measured concentration profiles of drugs in the plasma (observation) and model the calculated drug concentration profiles in the mixing chamber in the MPS (prediction). Eight drugs were selected as a training set. The authors also took additional steps for normalizing the *in vivo* and *in vitro* concentration profiles to account for differences in the dose and bioavailability. The normalized concentration is a dimensionless number, taking into account the drug bioavailability, volume of distribution, and drug dose. The authors explain that this normalization method allows direct comparison between the *in vivo* and *in vitro* concentration profiles, since they represent drug concentrations observed for a unit concentration in a unit distribution volume.

Using this algorithm, fitted design parameters were medium volumes for each compartment and the mixing chamber, and the luminal flow rate to the kidneys (in the case of the second MPS). It should be noted that the mixing chamber volume was fixed in the case of the second MPS, due to the practical reason of reducing the number of fitted parameters. Also, limits on the range of design parameters were imposed prior to the parameter fitting, to eliminate the possibility of physically impossible design parameters. The performance of the proposed scaling approach was quantitatively evaluated by comparing the simulated values of area under the normalized concentration curve (termed by the authors as AUNC) with those simulated from two other scaling methods, direct scaling and allometric scaling methods. While the direct and allometric scaling methods predicted AUNC values that are orders of magnitude lower than the *in vivo* AUNC, multifunctional scaling methods yielded AUNC values more comparable to *in vivo* values. A notable achievement of this paper is that after obtaining the design parameters with a training set of eight drugs, the accuracy of the MPS was evaluated with a test set of five drugs, and the authors were able to verify that the model prediction showed a reasonable agreement with known PK of the five drugs. This result shows that this multifunctional approach is valid at least for designing a multiorgan MPS for predicting the PK of drugs, although whether it is valid for designing MPSs for different purposes remains to be examined. We have summarized the main principles and pros and cons of different scaling methods in Table I.

**TABLE I.** Summary of different scaling methods.

Methods	Direct scaling	Residence time-based scaling	Allometric scaling	Functional scaling	Multifunctional scaling
Main principles	Multiplication of organ sizes by a scaling factor	Match the fluid (blood) residence time for each organ	Physiological parameters should follow allometric power laws at microscale	Define major functional parameter for each organ	Specify multiple objective parameters and numerically derive design parameters
Pros	• Very simple	• Ensures physiologically realistic dynamics between organs	• Plenty of literature sources for allometric relationship for various parameters	• Mathematically robust and easy to apply once data is provided	• Works well for a specific purpose (for example, PK study)
Cons	• Likely to cause imbalance between organ functions at microscale	• Each organ module should have physiological level of intrinsic activity	• Allometric scaling law may not hold at microscale	• Issues with organs with multiple functions	• Can be mathematically complex when the system becomes larger
	• Ignores flow rates or circulation time	• Mass transfer within the tissue needs to be considered	• Often requires further refinement by considering cell number, flow rates, etc.	• Difficult to define quantitative parameters for some organ functions	

## III. INTERPRETATION OF MPSs

Interpretation of experimental data obtained from multiorgan MPSs and translation of the data to *in vivo* requires appropriate mathematical modeling platforms. There are a number of existing modeling platforms used in pharmacology and in the pharmaceutical industry that could be adapted for this purpose. In particular, PBPK (physiologically based pharmacokinetic)–PD (pharmacodynamic) modeling technique has been successfully applied to analyze and predict the behavior of MPSs.^19,23^ More recently, use of the systems pharmacology approach for *in vitro in vivo* (IVIV) translation has been proposed.^37^ Here, we summarize recent examples of using mathematical modeling platforms together with the experimental approach using MPSs, with an emphasis on the technical aspects of the modeling methods.

### A. Empirical PK modeling

Pharmacokinetics (PK) is a study of time-dependent drug concentration in the body after exposure, and PK modeling is frequently used during the drug development process for dose adjustment, optimization of drug formulation, and prediction of toxicity and efficacy. Different types of PK models exist, with varying degrees of complexity. One of the simplest forms of a PK model is one- or two-compartment models, which assume the body as a single compartment or two interconnected compartments. Solving mass balance equations on the compartment model yields determinate solutions with exponential terms, which describe the time-dependent concentration profiles of a drug. Since a multiorgan MPS is basically a collection of multiple chambers (compartments) with interconnected flow, this simple compartment model can be directly applicable to the analysis of a multiorgan MPS.

Ouattara *et al.* demonstrated using compartment models how to analyze the PK data on benzo[a]pyrene obtained from a static well-plate system and a dynamic perfusion system, both containing Caco-2 and HepG2 cells to mimic absorption in the small intestine and hepatic metabolism, respectively.^40^ Mass balance equations describing the two systems were set up and solved, and the model fits for the two systems were compared with experimental data. Although the perfusion system used in this study is in a relatively simple and primitive form (perfusion was introduced to the conventional transwell culture platform), it is a clear example of how a simple compartment PK model can be combined with an experimental approach using a multiorgan MPS.

Prot *et al.* demonstrated a more advanced form of using compartment PK models with a multiorgan MPS to study the PK of paracetamol.^41^ A microfluidic MPS with Caco-2 (intestine), HepG2, and primary hepatocytes (hepatic metabolism) was used to study the first-pass metabolism of paracetamol, which was coupled with a mathematical model to estimate the intrinsic clearance parameters of the intestine and the liver. The mathematical model used in this study consisted of seven compartments (accounting for four medium reservoirs, hepatic, intestine compartments, and tubing between the compartments). Another notable distinction in this study is that an additional mathematical model was used for extrapolation of the *in vitro* hepatic intrinsic clearance rate to *in vivo* clearance parameters. Two different models (well-stirred and parallel tube models) were compared, along with using a scaling factor based on hepatocellularity per unit mass of liver and body mass for conversion from *in vitro* to *in vivo*. The hepatic clearance and availability parameters that were predicted from the experimental data coupled with mathematical models showed reasonable agreement with the corresponding *in vivo* values from literature.

This study by Prot *et al.* shows that coupling of a multiorgan MPS with traditional compartmental PK modeling technique may be useful for predicting *in vivo* PK parameters, but further validation with a set of different drugs (preferably drugs with different physical/chemical and PK characteristics) is necessary. In fact, employing a similar approach with different drugs, omeprazole and phenacetin, resulted in less satisfactory agreement with known *in vivo* PK parameters.^42^ Investigation into the possibility of using more mechanistic models to better account for the PK of different drugs in the MPS may contribute to improving the accuracy of the prediction by this approach. In addition, expansion of this approach to account for the action of drugs, that is, pharmacodynamics (PD) of drugs, will yield a more robust and useful MPS-modeling platform, as illustrated in Sec. III B.

The effect of hepatic metabolism on the cardiotoxicity of drugs was investigated using a multiorgan MPS.^17^ In this study, computational fluid dynamics simulation was used to predict the shear stresses and mixing profiles in each organ chamber. Conversion of a parent drug to a metabolite was modeled using a two-tier process, incorporating the uptake of a drug by hepatocytes, and conversion of a parent drug to a metabolite inside hepatocytes. The transport of a drug into the cells and the conversion process were modeled using the Michaelis-Menten kinetics model with constants being estimated from experiment. This two-tier model is more detailed than previously described PK models, since it simultaneously accounts for the transport and chemical conversion. The transport of a drug and its metabolite from the liver to other organs, such as the heart, was not explicitly modeled, which can be explained by the assumption that the system is not limited by the flow between different organs. Electrical and mechanical functions of cardiac tissues were examined under the influence of a drug and its metabolite. This study demonstrates that the pharmacological effect of a drug and its metabolite can be observed and analyzed by a combined experimental and mathematical modeling approach.

### B. Mechanistic PBPK-PD modeling (systems pharmacology approach)

Compartment PK models, although relatively simple, provide useful platforms for analyzing multiorgan MPSs and extracting key PK parameters from experimental data. However, as the multiorgan MPS becomes more advanced, including more organs and more complex action of drugs in the system, more mechanistic mathematical models are needed. Physiologically based pharmacokinetic (PBPK) models can provide a mechanistic basis, since they are based on the actual physiology and anatomy of the human body, and hence can better represent the mechanism of action the drugs elicit in the MPS.

Shuler *et al.* has published several papers examining the possibility of coupling PBPK models with multiorgan MPSs.^43^ In a paper published in 2008, Tatosian and Shuler compared the PK profiles of an anticancer drug doxorubicin (DOX) and its metabolite, doxorubicinol (DOXOL), predicted in the MPS with that predicted in the human body, by building a PBPK model for both MPSs and humans.^23^ Distribution profiles of DOX and DOXOL in different tissues resulting from 1 *μ*M DOX in the MPS and 100 g/m^2^ of DOX in human were compared, showing that the area under the curve (AUC) prediction is of the same order of magnitude. It is not certain whether the dose of 1 *μ*M in the MPS was comparable to the dose of 100 g/m^2^ in human, so a direct comparison of concentration profiles might not be possible. However, this study demonstrated a primitive form of coupling a PBPK model with a multiorgan MPS. Also, the MPS was scaled and designed on similar principles introduced in earlier paper,^6^ and it is possible that the hepatic conversion in the liver chamber was not realistic since a cell line was used in this study.

Pharmacokinetics (PK) is the study of concentration of drugs after administration and pharmacodynamics (PD) is the study of the effect of drugs. PK or PBPK models are based on the mass balance of drugs and their metabolites in each organ (or compartment), and PD models are based on empirical or mechanistic models describing the action of drugs at the target site. Coupling of PK and PD models has been frequently attempted, as it can be used to predict the pharmacological effect of a drug based on the administered dosage.^44^ Since it is possible to construct a PBPK model of a multiorgan MPS, it should be possible to couple this model with a PD model that describes the pharmacological effect of a drug in the MPS. In a paper published in 2010, Sung *et al.* developed a three-organ MPS (liver, tumor, and marrow) for testing the efficacy and toxicity of an anticancer drug, 5-fluorouracil (5-FU), and the corresponding PBPK-PD model of the MPS. In this study, the PD model describes the response of tumor cells to 5-FU, by assuming a number of different states (leading to complete cell death), where the transition from one state to the next state was defined by numerically fitted parameters. This early, proof-of-concept study showed that a coupled PBPK-PD can be used as a mathematical modeling platform for analyzing the action of drugs in a multiorgan MPS, as well as for gaining some insight into the action of drugs by extracting numerical parameters.

A similar approach was taken in a more recent study, where a two-organ MPS (liver-tumor) was coupled with a PK-PD model describing the anticancer effect of a flavonoid, luteolin.^45^ A notable conclusion from this study was that the PK-PD model of the MPS helped the authors to provide an explanation for discrepancies between the efficacy of the drug predicted from a conventional multiwell platform experiment and the actual efficacy observed in the MPS. The observed efficacy was considerably lower in MPSs than what was anticipated from the multiwell experiment, and by looking into the concentration profiles of luteolin and its metabolites, the authors were able to conclude that the hepatic conversion and tumor cell-killing action were occurring simultaneously, which caused the actual “working concentration” of the active drug to be lower than expected. Again, this study is a simple but important demonstration of how PK-PD models can provide a detailed account of what is happening inside the MPS, once we have a relatively mechanistic model of the system.

A systems pharmacology approach to MPS development and utilization was recently reported,^46^ which is conceptually similar to the PK-PD modeling approach mentioned above,^19^ but showed a technically more advanced approach. A single liver/immune MPS, containing human hepatocytes and Kupffer cells, was used to study the PK of hydrocortisone (HC) and the inflammatory response to lipopolysaccharides (LPS). A mechanistic model describing the PK of HC and the inflammatory reaction by LPS was constructed based on a single MPS, which was then expanded to the theoretical study of a four-organ MPS (liver, kidneys, gut and a hypothetical target PD organ). The authors verified that the mechanistic PK model for HC which includes the mechanism of HC binding to human serum albumin (HSA) was able to predict the PK of HC in both cases of low and high concentrations of HSA. Similarly, a semimechanistic model of inflammatory response to LPS, including the binding of LPS to Toll-like receptor 4 (TLR-4), LPS-TLR-4 complex internalization, TLR4 recycling, and TNF-α and IL-6 production by Kupffer cells, was able to predict the liver inflammatory response to LPS, as well as desensitization by subsequent doses. Such findings in this study demonstrate that having a detailed, mechanistic model of the MPS system based on the knowledge of biological mechanisms could improve the accuracy and utilization of the MPS. Furthermore, the authors combined the mechanistic liver/immune model with three more organs (gut, kidneys, and a target PD organ) and performed a sensitivity analysis on various design and operation parameters, from which they were able to draw some important insights about what kinds of parameters had strong effects on pharmacological outcomes. For example, the time required for the concentration of a chemical entity in the target PD organ to reach 80% of that in the mixing chamber (t_mixing,80_) was strongly dependent on the mixing chamber outlet flow and the target PD organ volume. These examples of combining PK-PD models (or systems pharmacology models) demonstrate the usefulness of such approaches, even at semimechanistic levels. A more detailed knowledge of the biological mechanisms in study will help researchers construct a more mechanistic model, which will then help improve the MPS.

## IV. FUTURE DIRECTIONS AND CONSIDERATIONS FOR MPS DESIGN AND INTERPRETATION

**FIG. 3.**
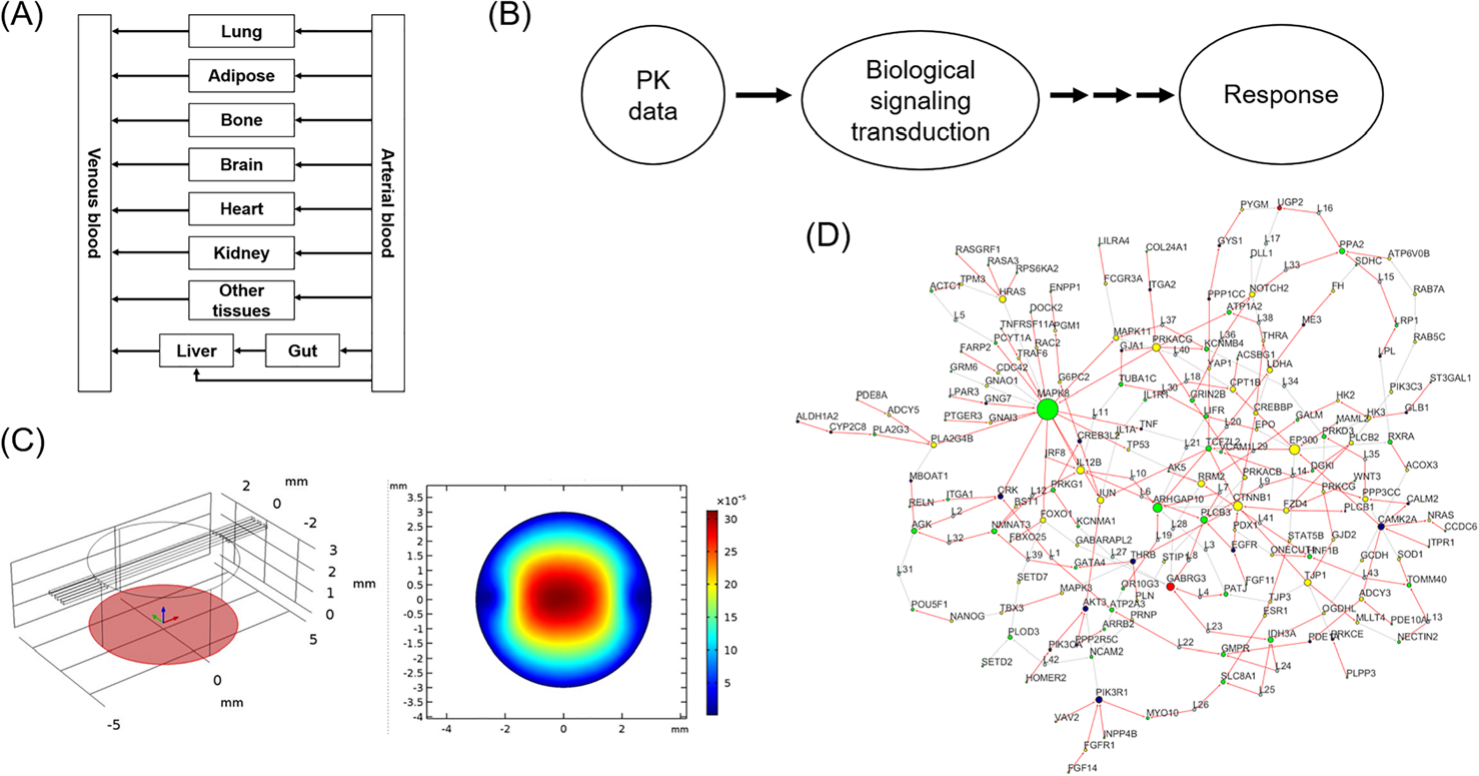
Types of mathematical models that can be used with MPS platforms. (a) pharmacokinetic (PK) models, (b) pharmacodynamic (PD) models, (c) fluid dynamics and transport models, and (d) biological network models. Reprinted with permission from Palluzzi *et al.*, PLoS One, **12**(10), 0185797 (2017). Copy right 2017 Palluzzi *et al.*, licensed under a Creative Commons Attribution (CC BY 4.0) license.^47^

Proof-of-concept studies for integrating multiple organs into a single device emerged in the 2000s. Earlier devices were based on rough calculations of chamber volumes and cell numbers, rather than accurate scaling of different organ sizes and flow rates between them. During the last five years, more rigorous approaches for designing a multiorgan MPS have been introduced. Some of them are extended from traditional allometric scaling laws across different organisms,^33,36^ some are based on chemical engineering principles and focus on mass balances and reaction kinetics,^6,7,24^ and more recently, multivariate optimization of parameters have been proposed.^39^ These studies prove that it is possible to design a multiorgan MPS for at least partially reproducing human physiology for a specific study. Interpretation of experimental data from a multiorgan MPS and extrapolation of the results to humans have been attempted with some success. Mathematical techniques that have been used in pharmacology or biology have been adapted successfully, but more extensive validation studies are needed to prove the usefulness of multiorgan MPSs in the drug development process. As can be seen in the literature described in this paper, coupling of *in vitro* MPSs with *in vivo* human can be beneficial on both ends, with the help of various available mathematical modeling tools. Figure 3 shows various kinds of mathematical modeling platforms that are widely used in the pharmaceutical science field, such as pharmacokinetic (PK) and biological network modeling.^47^

**FIG. 4.**
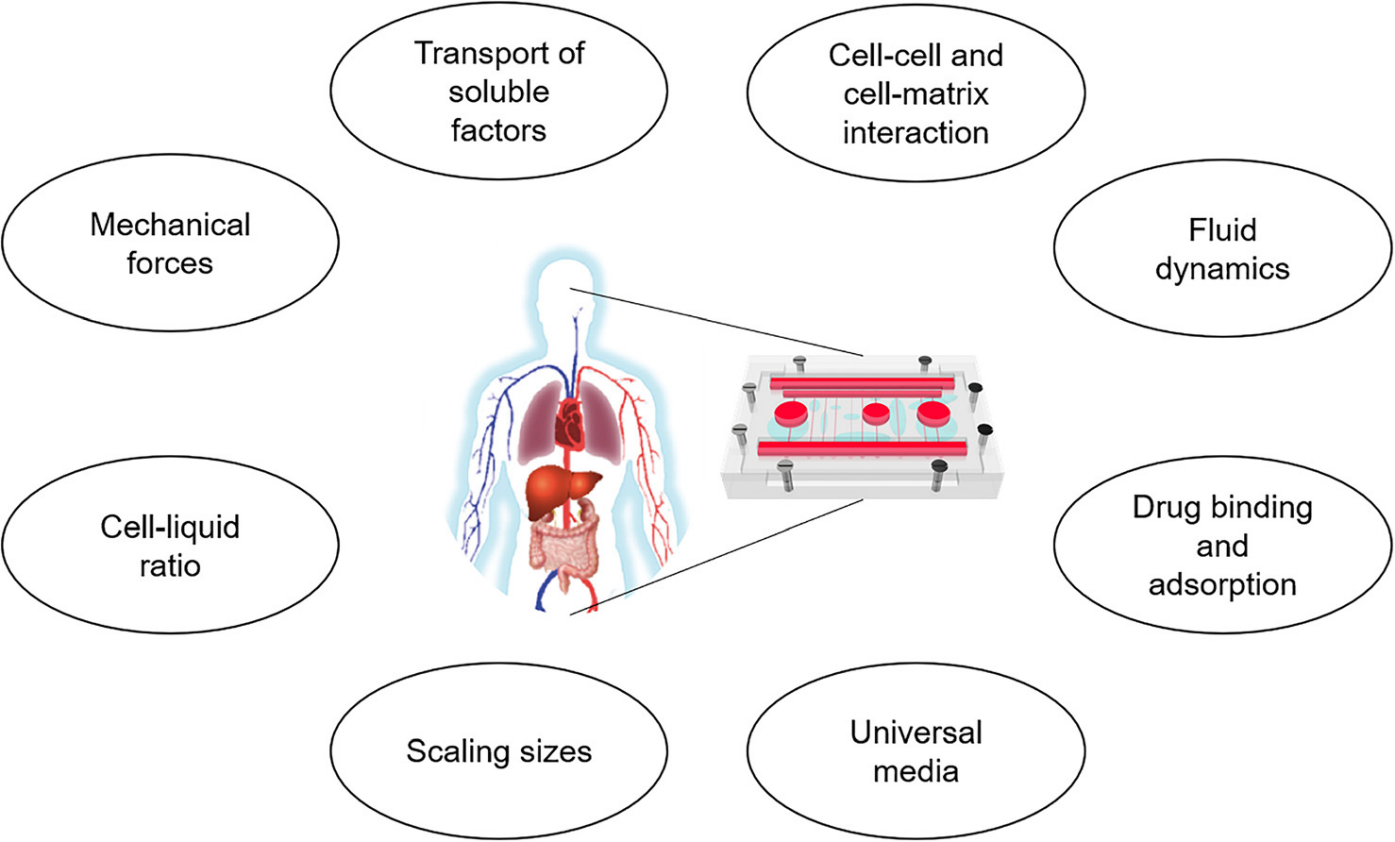
Considerations for building physiologically realistic multiorgan microphysiological systems.

To realize multiorgan MPSs for more comprehensive studies, it is necessary to simultaneously mimic the diverse aspects of human physiology, which depends on overcoming the remaining challenges (Fig. 4). As explained in Sec. II A, there are several aspects of the *in vivo* microenvironment that have not been realized. (1) The transport phenomena in MPSs are generally neglected or simplified. For example, cells in an MPS may be exposed to too low or too high concentrations of oxygen, when compared to the *in vivo* situation. Commercially available simulation software for fluid dynamics or mass transport can be helpful by providing detailed mathematical models of MPSs. (2) The issue of cell-to-liquid ratio has been brought up by several researchers,^6^ but realization of an MPS that faithfully meets this criterion has not been developed. This is particularly difficult, since the correct distribution of cells and liquids also needs to be considered, not only the overall ratio. More refined design of MPSs, often with the aid of mathematical modeling tools, to achieve a physiologically realistic ratio and the distribution of cells and liquids throughout the system, is needed. (3) The presence of an extracellular matrix (ECM) is an essential element in almost every organ and tissue, and different tissues have different requirements for the surrounding ECM. For example, the physiochemical properties of the ECM of the skin and the brain can be different. (4) A significant portion of a drug in the plasma binds to nonspecific proteins, which affect the PK and PD of the drug. It is possible that the binding kinetics of the medium in an MPS are different from that in the human blood plasma and need to be quantified. It is possible to build a PK model that accounts for the drug binding kinetics. When combined with experimental data, it should be possible to quantify the binding kinetics in the MPS, which can be accounted for when extrapolating to human data. (5) Some tissues are exposed to a variety of mechanical cues, such as fluidic shear, peristaltic motion, and contractile forces, or a combination of them. Each tissue needs to be exposed to appropriate amount and combination of such stimuli. Many of these stimuli are quantifiable and can be experimentally measured, which can be compared with predictions by mathematical models. (6) A widely known challenge is the issue of formulating the common medium, or artificial blood, that supports both the growth and the differentiation of not only one cell, but all cells from different types of tissues. We have seen some success in supporting two to four cell types in a system,^4,5^ but it becomes more challenging as the number of cell types in a system grows. The development of a chemically defined, serum-free common medium is a plausible solution, but it needs to be tested for a larger number of cell types.^48^ (7) Another complication is potential adsorption of medium components on the device, particularly devices made of PDMS (polydimethylsiloxane). This problem can be partially reduced by minimizing the exposure to inner surfaces and devising pumpless systems to remove tubing from the system,^45^ modifying the surface with less adsorptive properties,^49^ or substituting biocompatible thermoplastics for other materials such as PDMS.^50^ This adsorption phenomena can be incorporated into a mathematical model of MPS, similar to the protein binding process, to get a better view of the processes in an MPS. Several commercial modeling software are available. The fluid dynamics and transport phenomena within the MPS can be studied with modeling tools such as COMSOL Multiphysics® software (Burlington, MA, USA). There are also several commercial PK-PD modeling tools, which can be easily adapted to MPSs, such as PK-Sim (Bayer, Leverkusen, Germany), Simcyp (Certara, Princeton, NJ, USA), and MATLAB/SimBiology (Mathworks, Natick, MA, USA). (8) Finally, mathematical modeling platforms for multiorgan MPSs need further improvement and validation, as the systems become more complicated. State-of-the-art technologies in information technology, such as artificial intelligence and machine learning techniques, may be combined with MPS technology in future, as it has already been demonstrated in the case of using deep learning for automated analysis of vascularization images,^51^ or using Bayesian algorithms for parameter estimation.^52^

The recent progress in the development of single organ MPSs and multiorgan MPSs seems promising, in particular, with active movement toward commercialization in collaboration with the pharmaceutical industry.^53^ A combination of *in vivo, in silico*, and *in vitro* model platforms will enable wider applications for each model, and data obtained from both *in vitro* MPS platforms and *in vivo* platforms (human or animals) can be complementary to each other, and can also be used as input for *in silico*, mathematical model platforms (Table II). Development of hardware for MPSs should be accompanied by the corresponding development of mathematical modeling techniques for designing and interpreting the systems. Adoption of existing techniques or principles to MPSs is desirable and practical,^43,46^ while improvements to MPSs with specific purposes are still needed to achieve a wider acceptance and for application in the pharmaceutical industry.

**TABLE II.** Comparison of *in vivo* (human or animals), *in silico* (mathematical models), and *in vitro* (MPS) platforms. Different models can be used together for same applications, and data from different models can complement each other.

	*In vivo* (human or animals)	*In silico* (mathematical models)	*In vitro* (MPS)
Applications	• Personalized medicine	• Drug dosing and scheduling	• Hypothesis testing
	• Diagnosis and detection	• Designing MPS	• Drug screening (toxicity and efficacy)
	• Preventive medicine	• Hypothesis testing	• Study mechanism
Available data	• Biomarkers (blood, urine, and tissue samples)	• Concentrations of metabolites and drugs	• Concentrations of metabolites and drugs
	• Tissue and organ functional markers	• (Local) concentrations of cytokines/soluble factors	• (Local) concentrations of cytokines/soluble factors
	• Imaging data (X-rays, CT, MRI)	• Transport phenomena	• Transcriptome and proteome
	•PK parameters	• Fluid dynamics	•Mechanical functions (e.g., muscle contraction)
		•Time-dependent data	
			• Electrical functions (e.g., neuron activity)
			• Barrier functions (e.g., gut, kidneys, BBB)
